# Macrolactin A mediated biocontrol of *Fusarium oxysporum* and *Rhizoctonia solani* infestation on *Amaranthus hypochondriacus* by *Bacillus subtilis* BS-58

**DOI:** 10.3389/fmicb.2023.1105849

**Published:** 2023-02-23

**Authors:** Chitra Pandey, Deepti Prabha, Yogesh Kumar Negi, Dinesh Kumar Maheshwari, Shrivardhan Dheeman, Monika Gupta

**Affiliations:** ^1^Department of Basic Sciences, College of Forestry (VCSG UUHF), Tehri Garhwal, Uttarakhand, India; ^2^Department of Botany and Microbiology, Gurukula Kangri University, Haridwar, Uttarakhand, India; ^3^Department of Seed Science and Technology, School of Agriculture and Allied Sciences, HNB Garhwal University, Srinagar, Pauri Garhwal, Uttarakhand, India; ^4^Amity Institute of Microbial Technology, Amity University, Noida, Uttar Pradesh, India

**Keywords:** *Bacillus subtilis*, biocontrol, macrolactin, *mln* gene, scanning electron microscopy

## Abstract

Plant diseases are one of the main hurdles for successful crop production and sustainable agriculture development world-wide. Though several chemical measures are available to manage crop diseases, many of them have serious side effects on humans, animals and the environment. Therefore, the use of such chemicals must be limited by using effective and eco-friendly alternatives. In view of the same, we found a *Bacillus subtilis* BS-58 as a good antagonist towards the two most devastating phytopathogens, i.e., *Fusarium oxysporum* and *Rhizoctonia solani*. Both the pathogens attack several agricultural crops (including *amaranth*) and induce a variety of infections in them. The findings of scanning electron microscopy (SEM) in this study suggested that *B. subtilis* BS-58 could inhibit the growth of both the pathogenic fungi by various means such as perforation, cell wall lysis, and cytoplasmic disintegration in the fungal hyphae. Thin-layer chromatography, LC–MS and FT-IR data revealed the antifungal metabolite to be macrolactin A with a molecular weight of 402 Da. Presence of the *mln* gene in the bacterial genome further endorsed that the antifungal metabolite produced by BS-58 was macrolactin A. Pot trial conducted in the present study showed that seed treatment by BS-58 effectively reduced seedling mortality (54.00 and 43.76%) in *amaranth*, when grown in pathogen infested soil (*F. oxysporum* and *R. solani*, respectively), when compared to their respective negative controls. Data also revealed that the disease suppression ability of BS-58 was almost equivalent to the recommended fungicide, carbendazim. SEM analysis of roots of the seedlings recovered from pathogenic attack substantiated the hyphal disintegration by BS-58 and prevention of *amaranth* crop. The findings of this study conclude that macrolactin A produced by *B. subtilis* BS-58 is responsible for the inhibition of both the phytopathogens and the suppression of the diseases caused by them. Being native and target specific, such strains under suitable conditions, may result in ample production of antibiotic and better suppression of the disease.

## 1. Introduction

*Bacillus subtilis*, a Gram-positive, endospore former, is able to survive under adverse conditions, and is capable of synthesizing a vast array of beneficial metabolites. The potential of *B. subtilis* strains to produce a variety of secondary metabolites is known for decades. It is also known that at least 4–5% genome of any strain of the *B. subtilis* is responsible for the production of antimicrobial compounds (Stein, 2005). This species has also been identified as a good candidate for plant growth promotion and/or biocontrol of many plant diseases by different researchers (Pandey, 2018a; Caulier et al., 2019; De la Lastra et al., 2021). These organisms enhance plant growth and suppress plant diseases by different modes of action. The most common mechanisms are phytohormone production, nutrient solubilization and suppression of phytopathogens through various means, including the production of hydrolytic enzymes, siderophores, antifungal compounds, lipopeptides, antibiotics etc. (Negi et al., 2011; Pandey et al., 2018c; Hashem et al., 2019; Ku et al., 2021). *Bacillus subtilis* is widely known for the production of antimicrobial compounds and protection of different agricultural crops by suppressing phytopathogens (Chauhan et al., 2016; Guo et al., 2019; Chakraborty et al., 2020; Mulk et al., 2022). *Bacillus subtilis* being an environmentally benign biocontrol agent, its antimicrobial metabolites and other plant growth promoting traits are adequate to increase soil fertility, plant growth and disease suppression.

Secondary metabolites produced by *B. subtilis* are classified as ribosomally synthesized peptides (bacteriocins) and non-ribosomally synthesized peptides (lipopeptide and polyketide; Moyne et al., 2001). Antibiotics such as subtilosin, subtilin, ericin A, ericin S, mersacidin, TasA, sublancin, bacilysin, surfactin, plipastatin, bacitracin, fungycin, mycosubtilin, macrolactin, corynebactin, bacillomycin, amicoumacin etc. are known to be produced by *B. subtilis* (Moyne et al., 2001; Stein, 2005). Among these, macrolactin, a polyketide responsible for antimicrobial, anticancerous and other inhibitory activities is synthesized by the action of polyketide synthase (PKS; Schneider et al., 2007).

*Bacillus subtilis* BS-58, a promissing plant growth promoting bacterial (PGPB) strain was isolated from the non-rhizospheric soil sample collected from Salamkhet (Tehri, Garhwal; 78^o^24′37″E and 30^o^18′13″N) during our previous study on *amaranth* (Pandey et al., 2018c). Root-rot, stem decay and damping-off are prevalent in *amaranth* in this region and adversely affect crop health and its productivity. *Fusarium oxysporum* was found to be associated with root-rot and stem decay, whereas, *Rhizoctonia solani* was found responsible for root-rot and damping-off (Post-emergence). Both the pathogens are very common and responsible for heavy crop losses (~ 50–60%). Since, crop losses due to the attack of different pathogens and pests result in reduced food availability, they are considered as the major threats to global food security (Savary et al., 2019). *Amaranth* is one of the nutrient rich crops and is known as a good source of proteins, essential amino acids, macro and micronutrients (Shirani et al., 2017; Pandey et al., 2018b). Therefore, effective and eco-friendly management of such diseases has to be devised. Though, fungicides have been in use for the suppression of the pathogens, but, their long term and continuous use may cause lots of side effects on humans, animals and ecosystem (Pandey et al., 2018d; Fortunati et al., 2019). Therefore, biological approaches can be an effective and eco-friendly alternative for disease management. The endospore forming ability of *B. subtilis* gives it an upper edge to be used as abiocontrol agent and plant growth promoter.

In view of the above, the present study was focused on the assessment of biocontrol ability of *B. subtilis* BS-58 towards the two important phytopathogens (*F. oxysporum* and *R. solani*) of *amaranth* and identification of the antifungal metabolite produced by BS-58.

## 2. Materials and methods

### 2.1. Bacterial and fungal cultures

Basic details of *B. subtilis* BS-58 including, its isolation, identification, and its potential to increase plant growth and yield have already been published (Pandey et al., 2018c). The important traits of this strain include, phosphate solubilization, phytase production, siderophore production, IAA production and cold tolerance up to 5.0°C (Table 1). Both the fungal pathogens (*F. oxysporum* and *R. solani*) of *amaranth* were procured from the well-characterized repository of the Microbiology laboratory of College of Forestry (VCSG Uttarakhand University of Horticulture and Forestry), Ranichauri, Tehri Garhwal (Uttarakhand), India to conduct different experiments in this study. Out of these microorganisms, *Bacillus subtilis* BS-58 was maintained on nutrient agar medium (NAM) and the fungal pathogens were maintained on potato dextrose agar (PDA) slants at 4°C.

**Table 1:** Plant growth promotion and biocontrol potential of *Bacillus subtilis* BS-58.

Activities		Results	SEM	cd (*p* = 0.05)
P-solubilization efficiency^*^ (%)		165.0	0.47	1.84
Phytase production^*^		+		
Siderophore production efficiency^*^ (%)		78.0	1.29	5.04
IAA production^*^		+		
HCN production^*^		−		
Cold tolerance^*^ (up to 5°C)		+		
Antagonistic efficiency (%)	*Fusarium oxysporum*	68.25	0.42	1.65
	*Rhizoctonia solani*	64.50	0.84	3.29

* Data taken from Pandey (2018a).

+: Positive reaction, −: Negative reaction. cd: Critical difference. Sem: Standard error of the mean.

### 2.2. *In vitro* antagonistic activity

The antagonistic activity of *B. subtilis* BS-58 was carried out against both the phytopathogens (*F. oxysporum* and *R. solani*) using the dual culture plate technique (Skidmore and Dickinson, 1976). Briefly, the fungal discs (6 mm dia) were excised from fully grown 5 days old cultures of both the fungi and were placed at the center of another medium plate (containing NAM + PDA in 1:1) individually. Challenge inoculation of *B. subtilis* BS-58 was done on both sides of the fungal disc (2.0 cm apart from the disc). The plates were then kept for incubation at 27 ± 1°C for 3–5 days. Plates only with fungal growth (without challenge inoculation) were kept as control and per cent inhibition of fungal growth in dual culture plate was calculated over control plate by using the following formula:


$$\%\mathrm{Inhibition}=\frac{C-T}{C}X\;100$$

(where, C = Radius of fungal growth in control plate, T = Radius of fungal growth in dual culture plate)

### 2.3. Scanning electron microscopy

To understand the inhibitory action of bacterial cells on the growth of the fungal pathogens in dual culture plates SEM analysis was done by following the method of King and Brown (1983) with some modification. Briefly, small pieces of agar (~ 1 cm^2^) from the zone of interaction were excised from each plate and transferred to the well-dried interior surface of the lid of a glass Petri dish. Fungal discs were fixed overnight at 4°C in 4% glutaraldehyde in 0.05 M phosphate buffer (pH 7.3) and washed thrice (10 min each) in phosphate buffer. After washing, samples were serially dehydrated (thrice) in 70, 80, 90, and 100% ethanol (5 min at each step) followed by air-drying. Dried samples were mounted on stubs and coated with gold. These coated specimens were observed at 15 KV in a LEO 485 VP Scanning Electron Microscope and photographs were captured.

### 2.4. Inhibitory potential of cell-free supernatant

The broth medium was prepared by following the composition as described by Kumar et al. (2014) and sterilized in an autoclave. The broth was then inoculated with *B. subtilis* BS-58 inoculum and incubated at 27 ± 1°C for 72 h to reach in the exponential phase (3 × 10^9^ cfu ml^−1^). The cells were then harvested by centrifuging at 8000 rpm for 10 min at 4°C and the supernatant was filtered through a Millipore filter (0.22 μm) to make it completely cell free. The antagonistic activity of cell free supernatant (CFS) was assessed against *F. oxysporum* and *R. solani* by agar well method by loading 100 μl CFS in each well.

### 2.5. Determination of the nature of antifungal metabolite

The cell-free supernatant (CFS) was then evaluated for its stability against heat and proteinase K treatment. The heat stability of the culture supernatant was assessed at two different temperatures (70 and 100 ± 1°C) for 20 min in a water bath following the method of Deraz et al. (2005). However, proteinase K (100 μg ml^−1^) treated sample was incubated at 37 ± 1°C in a water bath for 30 min. All the treated cell-free supernatant samples were then loaded into agar wells (100 μl in each well) made in assay plates (2.0 cm apart from the fungal disc) for the determination of antifungal activity. Development of a zone of inhibition (if any) was observed after incubation at 27 ± 1°C for 3–5 days.

### 2.6. Purification and identification of the antifungal metabolite

Purification of the bioactive molecule (antifungal metabolite) from CFS was done using thin layer chromatography (TLC) guided column chromatography as described below.

#### 2.6.1. Thin layer chromatography

Thin layer chromatography was performed on silica plates using different solvent systems (Supplementary Table S1) to select the most appropriate solvent system (mobile phase) for the separation of the antifungal metabolite. The plates were then kept in an iodine chamber to develop the spots of the compound.

#### 2.6.2. Column chromatography

Purification of the bioactive metabolite from the CFS was done by TLC guided column chromatography. For this, CFS having antifungal activity was first concentrated at 55°C using a rota-evaporator. The concentrated fraction was mixed thoroughly with silica in 1:3 and dried to prepare the loading sample. Column was prepared using silica (60–120 mesh size) and packed in the respective solvent. The sample was then placed at the top of the column and run with mobile phase (Ethyl acetate: Methanol). To elute the bioactive molecule, polarity of the solvent was increased by 5 % after each cycle. The column cycles were run with 500 ml of each solvent and elute size was kept 25 ml. Purity of the active compound in the collected elute was confirmed by obtaining a single spot on TLC plate. After identifying the elute containing pure compound, the solvent was evaporated and antifungal activity was re-assessed by agar-well method. Identity of the bioactive compound was then resolved on the basis of LC–MS and FT-IR analysis as described below.

#### 2.6.3. Liquid chromatography-mass spectrum analysis

Liquid chromatography-mass spectrum, with an electrospray ionization (ESI) interface, was used to determine the bioactive compound(s) in *the active fraction*. LC–MS analysis of the active fraction was performed on a UPLC (Ultra performance liquid chromatography) system, attached to an ESI interface and ACCUCORE-Mass spectrometer (Bruker Daltonic, CA, United States). MS spectra were collected in the scan range 150–1,000 m/z. Analytical chromatographic separations of the *active fraction* were carried out *via* a C18 100 × 3 column (50 × 2.1 mm, 1.7 μm; Thermo Fisher Scientific). The mobile phases used in this study were (A) acetonitrile + water (5:95), (B) acetonitrile, (C) methanol, and (D) water + formic acid at a fow rate of 0.3 ml min^−1^. Five microliters of the sample was injected, and the solvent was run by gradient elution. The positive ion mode of ESI–MS was used to acquire the mass spectra.

#### 2.6.4. Fourier-transformed infrared spectroscopy

Fourier-transformed infrared spectra of the pure compound were recorded on 8400S, FT-IR spectrometer (Spectrum GX) equipped with a mercury-cadmium-telluride (MCT) detector and cooled with liquid nitrogen. The extract from pure fraction was compressed into a thin pellet and analyzed at wavelengths of 400–4,000 cm^−1^. The analysis of FT-IR spectra was carried out by using OPUS 3.1 (Bruker Optics) software (Davis and Mauer, 2010).

### 2.7. Detection of the *mln* gene

For this, total genomic DNA of *B. subtilis* BS-58 was isolated by the phenol-chloroform extraction method as described by Ausubel et al. (1999). The presence of the *mln* gene in *B. subtilis* BS-58 was confirmed by its specific amplification using a pair of gene specific primers (MLN-C1 ATGCTGTTGCAGGACATAGTC and MLN-C2 TAGTCAGAATGTTTCCAGGACC; Schneider et al., 2007). Reaction mixture (100 μl) for the amplification was prepared containing 25 ng DNA, 1 × PCR buffer, 400 ng of each of the primers, 2.5 mM of each of the dNTPs, 0.3 U Taq polymerase. The PCR amplification was carried out with 35 cycles of initial denaturation (at 95°C for 3 min.), denaturation (at 94°C for 1 min.), annealing (at 50°C for 1 min.), synthesis (at 72°C for 2 min.), and extension (at 72°C for 7 min.). The amplicon was eluted from the gel and sent for sequencing at Biokart India Pvt. Ltd., Bengaluru, India. The sequence homology was studied by BLASTn search program. The sequence obtained was aligned by ClustalW using the MEGA7 software and the phylogenetic tree was constructed using the neighbor-joining method. The sequence was then submitted to NCBI by using Blanklt tool.

### 2.8. Pot trial for disease management

A pot-trial experiment (30 days) was carried out in pots (12″ dia) to evaluate the biocontrol ability of *B. subtilis* BS-58. The pots were filled with a pre-sterilized potting mixture containing sand, soil and farmyard manure (1:1:1). Seeds were moistened with sterile distilled water and coated with talc formulation (@ 10 g kg^−1^ seed) of *B. subtilis* BS-58. However, seeds for control set were moistened but did not receive the bacterial treatment. In case of the negative controls, soil was infested with the respective fungal pathogen (*F. oxysporum* and *R. solani*, individually) and seeds did not receive any treatment in these sets. Whereas, in positive control sets seeds were treated with carbendazim (@ 2.0 g kg^−1^ seeds) prior to sowing in pot soil infested with the respective fungal pathogen (*F. oxysporum* and *R. solani*, individually). Ten seeds per pot were sown at a depth of 1 cm. Germination of the seeds was recorded daily until all the seeds germinated in any of the pot. The other plant growth parameters were recorded at 30 day after sowing (DAS). The percent mortality of *amaranth* seedlings was calculated by following formula:


Seedling mortality%=Total number of dead seedlingsTotal number of seedlings germinatedX100

### 2.9. Post interaction events after pot trial

Scanning electron microscopy analysis was performed to understand the antagonistic effect of *B. subtilis* BS-58 on hyphal morphology. *Amaranth* seedlings showing characteristic symptoms (brown spots on stem and damping-off) were taken out from the respective pots. These stems and root samples were washed with sterile distilled water and dried before proceeding for SEM analysis. Dried samples were mounted on stubs and coated with gold. These coated specimens were observed at 15 KV in a LEO 485 VP Scanning Electron Microscope and photographs were captured.

### 2.10. Data analysis

The data recorded during the study was subjected to analysis of variance (ANOVA) using completely randomized design (CRD) to evaluate the significance by the magnitude of *F* value. Duncan Multiple Range Test (DMRT) was performed to compare the means by using SPSS Statics v26.

## 3. Results

### 3.1. *In vitro* assessment of antagonistic activity

**Figure 1:**
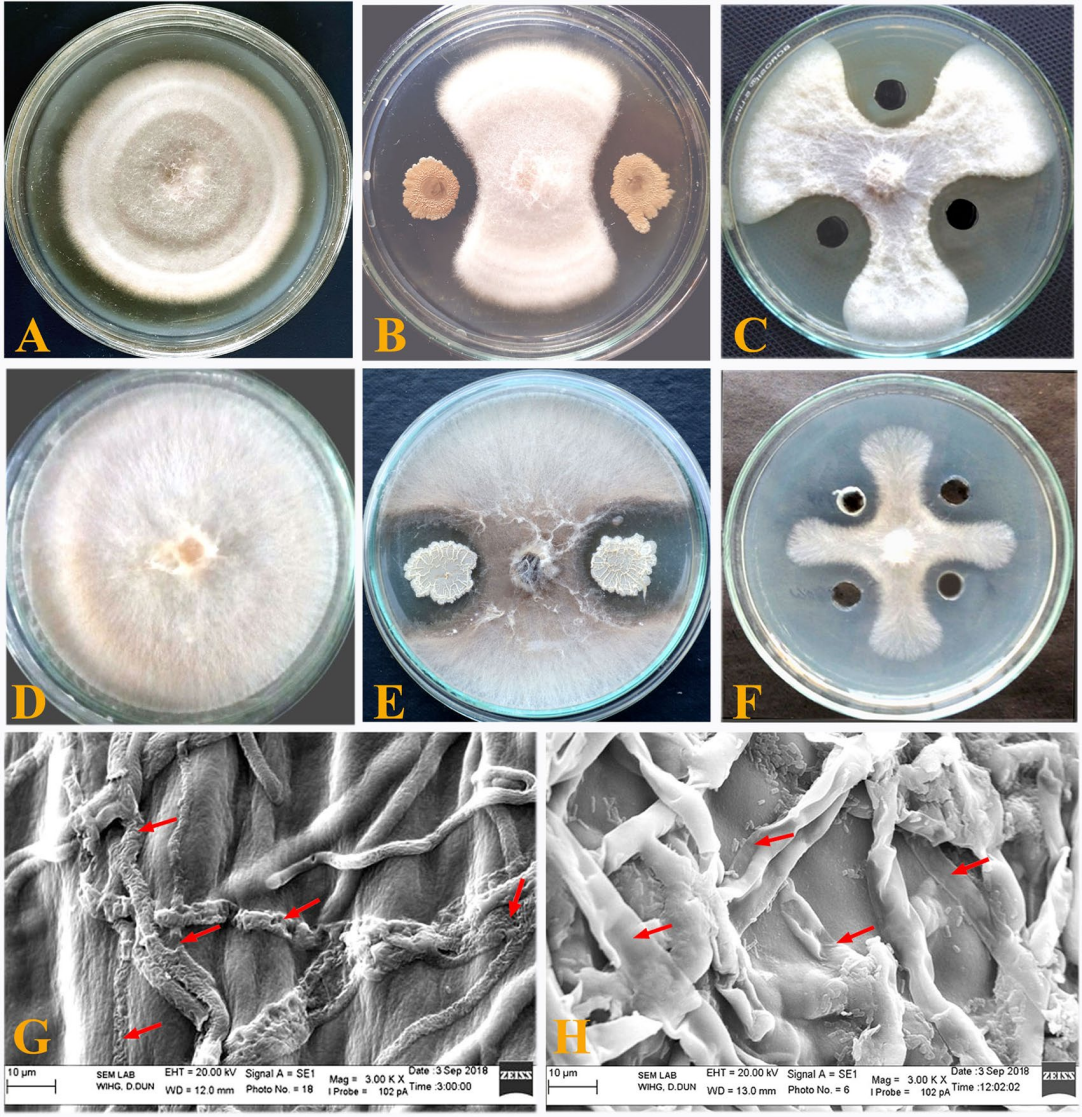
*In vitro* antagonistic activity of *Bacillus subtilis* BS-58 against both the fungal pathogens. **(A)**: Growth of *Fusarium oxysporum* in control plate; **(B)**: Inhibition of *F. oxysporum* by *B. subtilis*; **(C)**: Plate showing activity of elute (F7) against *F. oxysporum*; **(D)**: Growth of *Rhizoctonia solani* in control plate; **(E)**: Inhibition of *R. solani* by *B. subtilis*; **(F)**: Plate showing activity of elute (F7) against *R. solani*. **(G)**: Deformed mycelium (arrows) of *Fusarium oxysporum* upon interaction with *B. subtilis* BS-58; **(H)**: Deformed mycelium (arrows) of *Rhizoctonia solani* upon interaction with *B. subtilis* BS-58.

*Bacillus subtilis* BS-58 displayed good inhibitory potential against *F. oxysporum* and *R. solani* with 68.25 and 64.50% inhibition, respectively (Table 1; Figures 1A,B,D,E). Scanning electron micrographs showed deformities in hyphal morphology of *R. solani* and *F. oxysporum* in post-interaction events performed with samples taken from dual culture plates. These deformities included hyphal lysis, distortion, swelling, perforation, shrinkage and mycelial shredding in both the fungal pathogens (Figures 1G,H).

### 3.2. Antimicrobial activity of cell free supernatant and nature of the metabolite

Cell free supernatant of *B. subtilis* BS-58 collected after 72 h of incubation showed good antifungal activity against *F. oxysporum* (65.57%) and *R. solani* (61.66%). This was also interesting to note a good antifungal activity of *B. subtilis* BS-58 even after heat treatment at both of the temperatures (70 and 100°C) against *F. oxysporum* (62.76, 58.8%) and *R. solani* (59.33, 57.6%). However, some reduction in the antifungal activity against both the fungi (*F.* oxysporum: 28.9% and *R. solani*: 22.2%) after proteinase K treatment was recorded (Table 2; Figures 1C,F).

**Table 2:** Effect of various treatments on antifungal activity of cell-free supernatant (CFS) of *Bacillus subtilis* BS-58.

Pathogens	Treatment		Pathogen inhibition (%)	sem	cd (*p* = 0.05)
*F. oxysporum*	No treatment		65.57^a^	0.22	0.71
	Heat treatment	70^oC^	62.76^b^		
		100^oC^	58.8^c^		
	Proteinase K		28.96^d^		
*R. solani*	No treatment		61.66^a^	0.12	0.39
	Heat treatment	70^oC^	59.33^b^		
		100^oC^	57.6^c^		
	Proteinase K		22.24^d^		

Data presented in the table is the average of three replicates. Values in the table represented with different letters are significantly different (*p* < 0.05). cd: critical difference. Sem: standard error of the mean.

### 3.3. Purification and identification of the antifungal metabolite

Results of thin layer chromatography revealed the mixture of ethyl acetate and methanol (60:40) as the most appropriate solvent system for the separation of antifungal metabolite and therefore selected as the mobile phase for column chromatography. Among all the elutes collected from column chromatography, A4, A5 (100%), F1, F5, and F7 (55:45) showed inhibitory potential against *F. oxysporum* and *R. solani* (Table 3). All the active elutes were run on TLC plates to validate the purity of the bioactive compound.

**Table 3:** Activity of elutes and solvent system used for their separation in column chromatography.

Coloumn Elutes	Pathogen Inhibition (%)	
	*F. oxysporum*	*R. solani*
A4 (100% EA)	55.67^d^	54.11^c^
A5 (100% EA)	52.85^e^	51.76^d^
F1 (55% EA: 45% M)	59.17^c^	72.94^b^
F5 (55% EA: 45% M)	63.77^b^	72.94^b^
F7 (55% EA: 45% M)	71.90^a^	75.30^a^
sem	0.53	0.21
cd (*p* = 0.05)	1.65	0.66

Data presented in the table is average of three replicates. EA-Ethyl acetate; M-Methanol. Values in the table represented with different letters are significantly different (*p* < 0.05). cd: Critical difference. Sem: Standard error of the mean.

#### 3.3.1. Liquid chromatography-mass spectrum analysis

**Figure 2:**
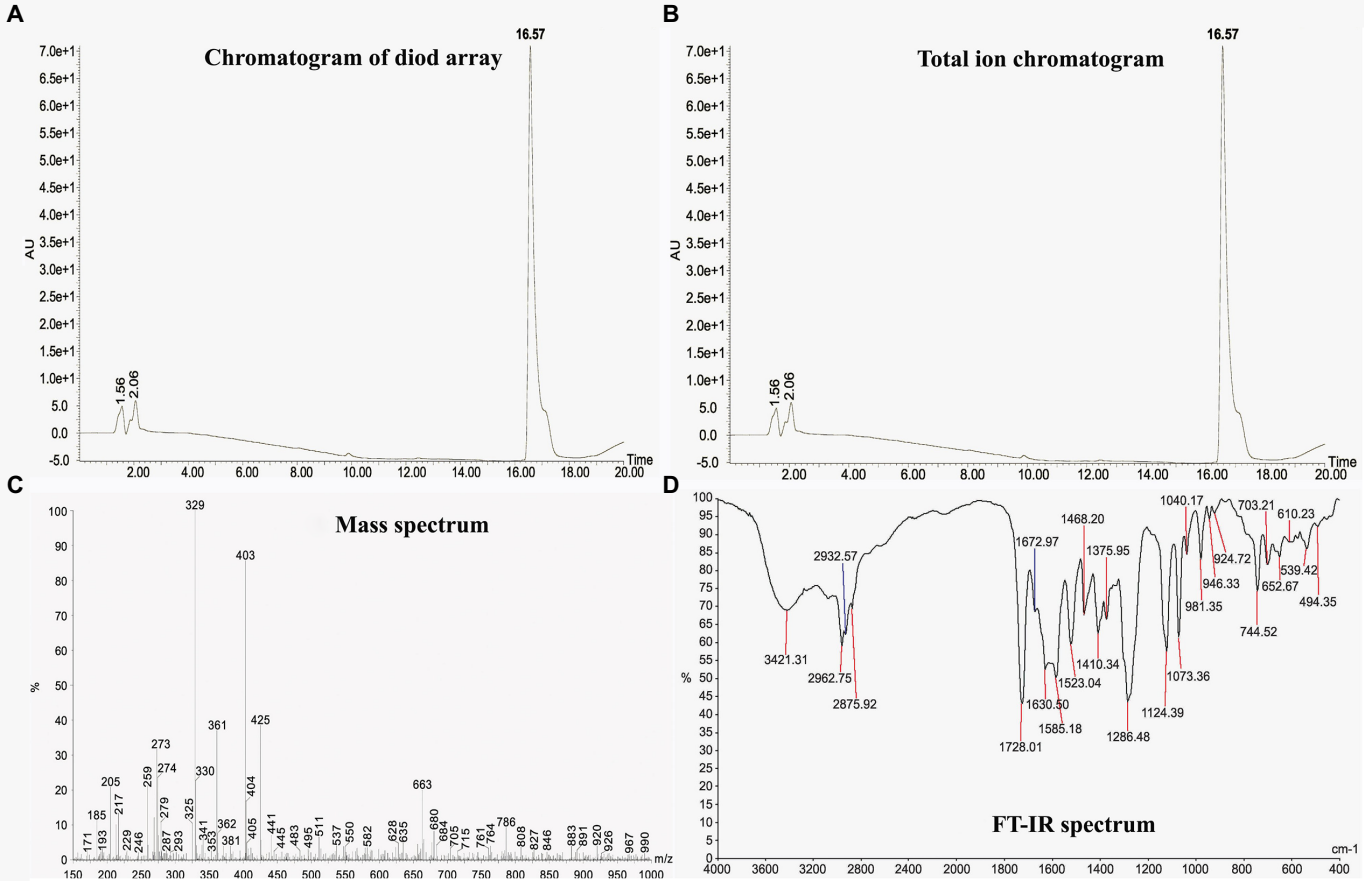
Liquid chromatography-mass Spectrum (LC–MS) and FT-IR analysis of the antifungal metabolite produced by *Bacillus subtilis* BS-58. **(A)**: Diode array chromatogram showing a major peak of the metabolite at 16.57 min; **(B)**: Total ion chromatogram showing two major peaks at 16.61 and 18.31 min suggesting the presence of cis and trans geometric forms of olifins in the metabolite; **(C)**: Mass spectra showing different peaks with different m/z value including strong peak of protonated metabolite (M + H) at 403 along with its sodium adduct ion (M + Na) peak at 425; **(D)**: FT-IR analysis showing different peak values for various functional groups including alcohol at 3421.31, acid at 1728.01, carbonyl at 1672.97.

Liquid chromatography of the most active elute (F7) showed a strong retention peak at 16.57 min in its diode array chromatogram for the active metabolite along with two small peaks at 1.56 min and 2.06 min (Figure 2A). Furthermore, appearance of one major peak at 16.61 min and another on 18.31 min in the positive electrospray scan suggests the presence of cis and trans geometrical isomers of olefins in the active metabolite (Figure 2B). A positive electrospray scan determined different peaks with different m/z (mass to charge ratio) value including strong peak of protonated metabolite at 403 along with its sodium adduct ion peak at 425 (Figure 2C).

#### 3.3.2. Fourier-transformed infrared spectroscopy (FT-IR)

Fourier-transformed infrared spectroscopy spectroscopy revealed different peaks representing different functional groups including, alcohol at 3421.31, alkane at 2932.57, 2962.76, 2875.92, and 1410.34, carbonyl at 1728.01 (Figure 2D). Combining the data received from LC–MS and FT-IR analysis, identity of the molecule was found to be macrolactin A with a molecular weight of 402 Da.

### 3.4. Detection of the *mln* gene in *Bacillus subtilis* BS-58

**Figure 3:**
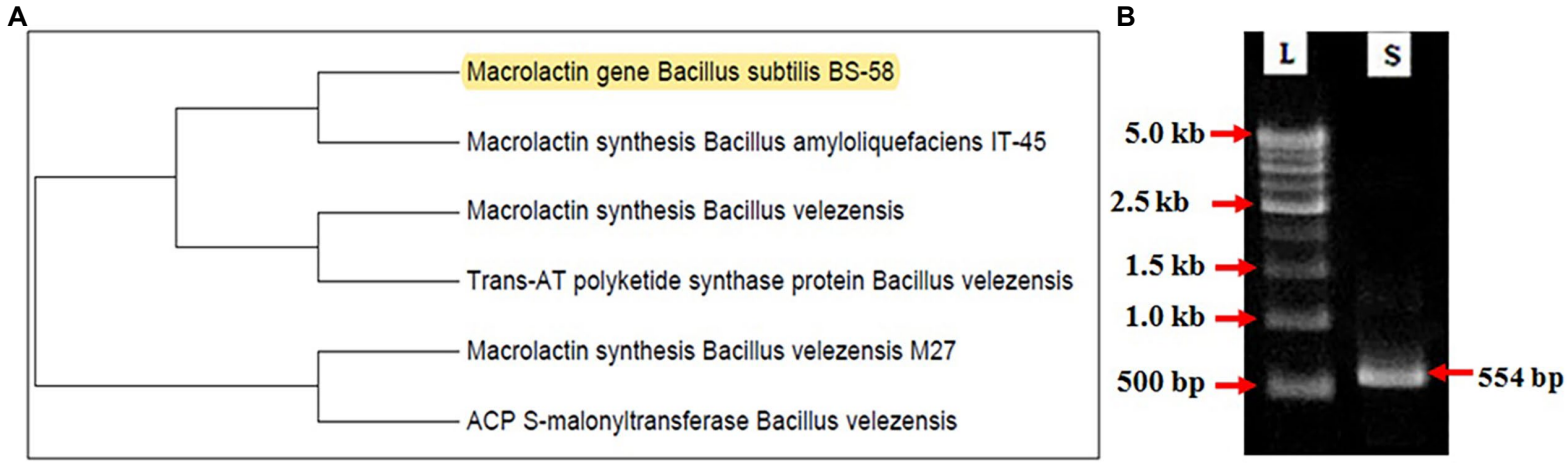
Identification of the *mln* gene in *Bacillus subtilis* BS-58. **(A)**: Figure showing relationship between the *mln* genes isolated from *B. subtilis* BS-58 and other *Bacillus* strains. **(B)**: Amplification of 554 bp fragment of *mln* gene [L-DNA ladder (500–5 kb); S-Amplified product of *Bacillus subtilis*].

The *mln* gene was isolated from *B. subtilis* BS-58 and identified by PCR amplifications using the gene specific primers. The DNA sequence retrieved from the amplicon (Supplementary Table S2) was submitted in gene bank with accession number MT726941. The sequence homology studied by BLASTn search program revealed 98.1% homology with the macrolactin genes available in the database at NCBI. Dendrogram showing the similarity of the *mln* gene isolated from *B. subtilis* BS-58 and other strains is presented in Figure 3A, and the specific amplification of the fragment is shown in Figure 3B.

### 3.5. Pot trial for disease management

**Figure 4:**
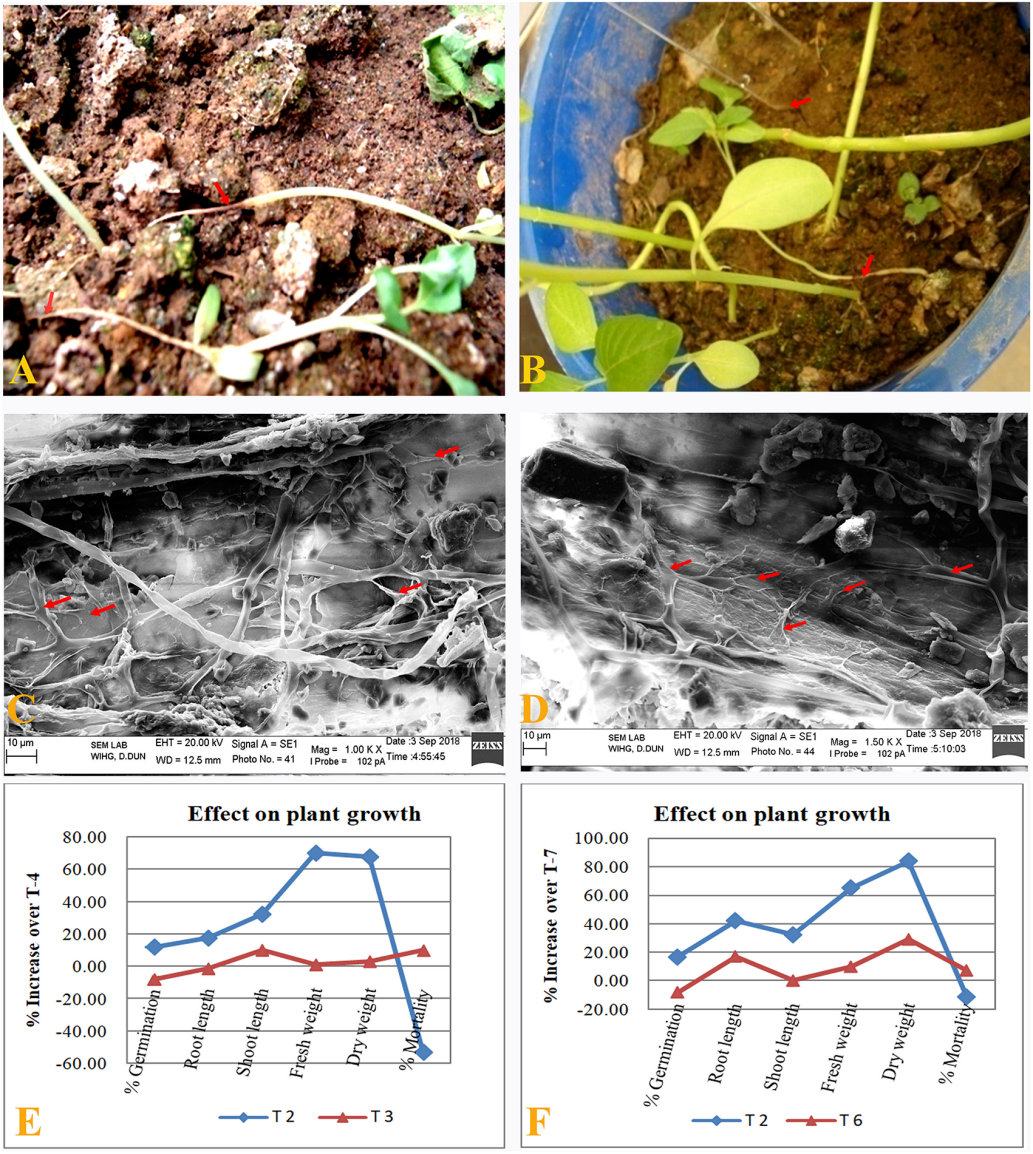
Disease suppression by *B. subtilis* BS-58 in pot assay. **(A)**: Seedlings grown in *R. solani* infested pot. **(B)**: Seedlings grown in *F. oxysporum* infested pot. **(C,D)**: SEM photomicrographs showing degradation and lysis of mycelia (note arrows) of both the pathogens (*R. solani* and *F. oxysporum*) by BS-58 under pot assay. **(E,F)**: Per cent increase in plant growth parameters calculated for different treatments over respective positive control (T-4 and T-7, respectively) [T-2: Seed treatment by BS-58; T-3: Seed treatment by BS-58 + Soil infestation with *F. oxysporum*; T-4 (Positive control: Soil infestation with *F. oxysporum* + Seed treatment by 0.2% carbendazim); T-6: Seed treatment by BS-58 + Soil infestation with *R. solani*; T-7: (Positive control: Soil infestation with *R. solani* + Seed treatment by carbendazim)].

Promising effects of seed treatment by *B. subtilis* BS-58 on seedling growth and disease suppression were observed in *amaranth* (Table 4). During this study, less seedling emergence as well as survival was observed in the pathogen infested soil (*F. oxysporum*: T-5, and *R. solani*: T-8; Figures 4A,B). Both of these treatments showed typical symptoms of infection of both the fungi including, root-rot, brown spots on stem and post emergence damping off. However, this was encouraging to note that no symptoms of infection were observed on seedlings grown out of the seeds treated with *B. subtilis* BS-58 in *F. oxysporum* infested soil (T-3). Whereas, *amaranth* seedlings grown in *R. solani* infested soil (T-6) and received seed treatment with *B. subtilis* BS-58 showed some early symptoms of infection (brown spots on stems). However, most of these seedlings recovered in the later stages of plant growth. Both of these treatments showed 17.85 per cent mortality. Increased shoot length, root length and other seedling growth parameters were recorded in uninfested sets (T-2 and T-4) over control (T-1). This was interesting to note that the bacterized seeds exhibited reduced percent mortality and improved growth of seedlings in pathogen infested soil. Maximum plant mortality was recorded in T-5 and T-8 (negative controls) infested with fungal pathogens (*F. oxysporum* and *R. solani*, respectively), where seeds did not receive any bacterial treatment (Table 4). However, minimum per cent mortality was recorded in T-2 (10.75%) followed by T-4 (14.81%).

**Table 4:** Effect of *Bacillus subtilis* on seedling growth and disease suppression.

Treatments	% Germination	Shoot length (cm)	Root length (cm)	Fresh weight (g)	Dry weight (g)	Mortality (%)
T1	86.67^a^	14.88^b^	20.34^c^	1.50^b^	0.52^b^	16.67^b^
T2	93.33^a1^	16.44^a^	26.00^a^	1.70^a^	0.58^a^	10.73^b^
T3	76.67^b^	15.40^b^	21.60^b^	1.01^d^	0.46^c^	17.85^b^
T4	90.00^a^	15.48^b^	18.85^d^	1.00^d^	0.35^d^	14.81^b^
T5	60.00^d^	13.66^cd^	16.66^f^	0.50^e^	0.21^e^	38.89^a^
T6	73.33^bc^	15.30^b^	20.75^c^	1.13^c^	0.41^c^	17.85^b^
T7	86.67^a^	14.53^bc^	19.35^d^	1.03^cd^	0.31^d^	15.27^b^
T8	66.67^cd^	13.10^d^	17.67^e^	0.60^e^	0.20^e^	31.74^a^
sem	2.89	0.29	0.22	0.04	0.02	3.84
cd (P = 0.05)	8.65	0.87	0.68	0.11	0.07	11.52

Data presented in the table is average of three replicates. T-1: No treatment; T-2: *B. subtilis*; T-3: *B. subtilis* + *Fusarium oxysporum*; T-4 (Positive control): *F. oxysporum* + 0.2% carbendazim; T-5 (Negative control-1): soil inoculated with *F. oxysporum*; T-6: *B. subtilis* + *Rhizoctonia solani*; T-7: (Positive control): *R. solani* + 0.2% carbendazim; T-8 (Negative control-2): soil inoculated with *R. solani*. Values in the table represented with different letters are significantly different (*p* < 0.05).

cd: critical difference. Sem: standard error of the mean.

### 3.6. Scanning electron microscopy

Effect of *B. subtilis* BS-58 treatment in the suppression of disease or restoration of health of the seedlings (after fungal infection) was also studied under the scanning electron microscope. A huge network of fungal mycelia (*R. solani*) was observed in the decayed seedlings of *amaranth* grown out of the untreated seeds, while inhibition of fungal mycelia by the swelling, fragmentation, lysis was observed in the seedlings survived from the fungal infection in BS-58 treated pots (Figures 4C,D).

## 4. Discussion

Extensive and sometimes inappropriate use of harmful agro-chemicals adversely affects the soil ecology and disturbs the environment, as well (He et al., 2008). Therefore, the use of microbial bioagents can be a chemical-free alternative to the conventional crop protection in agriculture and dependency on fungicides (Negi et al., 2017; Singh et al., 2022). Our study revealed that *B. subtilis* BS-58 could effectively suppress the growth of two destructive fungi, *F. oxysporum* (64.7%) and *R. solani* (73.3%). Our results get support from Zhu et al. (2020), who reported 67% inhibition of *F. oxysporum* f. sp. *niveum* by *B. subtilis* IBFCBF-4. Similarly, Hussain and Khan (2020) recorded 45% inhibition of *R. solani* (causal agent of black scurf disease of potato) by *B. subtilis*. The antifungal activity of *B. subtilis* BS-58 against both the fungal pathogens in our study might be due to the production of antifungal metabolite(s) or siderophore production those have been described as effective mechanisms of pathogen suppression by several researchers (Negi et al., 2017; Ku et al., 2021; Zhu et al., 2021). *Bacillus subtilis* BS-58 has already been reported with good colonization and plant growth promoting abilities in one of our previous studies (Pandey et al., 2018c). Therefore, good antifungal activity of BS-58 may protect the host plant from diseases caused by these fungal pathogens and simultaneously can reduce the dependence on fungicides being used for disease management.

In the present study, the loss of structural integrity of mycelia with hyphal swelling, lysis, digestion and perforation in both the fungi (*F. oxysporum* and *R. solani*) was observed in the SEM studies of the challenged fungal mycelia. Such deformities in hyphal morphology have been attributed to the production of antifungal metabolites by different biocontrol agents (Negi et al., 2011; Gomaa, 2012; Jimtha et al., 2016; Zhu et al., 2020). Similar findings were reported by Kaur et al. (2015) in post-interaction studies of *Alternaria alternata* and *B. vallismortis* R2. They observed shrunken, collapsed, empty hyphae, large depressions and loss of turgidness of *A. alternata* hyphae.

Cell free supernatant (CFS) of *B. subtilis* BS-58 was found inhibitory for *F. oxysporum* (65.57%) and *R. solani* (71.66%) in agar well diffusion assay. The results gets support from the study of Zhang et al. (2008), who found the CFS of *B. subtilis* B-FSo6 inhibitory towards *Aspergillus flavus* and suggested that the activity was due to the secretion of bacillomycin like compound by B-FSo6. Similarly, Kumar et al. (2012) showed the inhibition of *F. oxysporum* (50%), *Macrophomina phaseolina* (53.58%), *F. solani* (47.39%), *Sclarotina sclerotiorum* (47.69%) and *R. solani* (46.37%) by *Bacillus* spp. BPR7 and suggested that the antifungal activity might be due to the production of antifungal metabolites. Our results suggest that the antifungal metabolite is extracellular in nature and suppressing the fungal growth through diffusion in medium. Such metabolites when are diffused in rhizosphere may guard the crop from seed or soil borne pathogens.

Heat treatment of CFS of *B. subtilis* BS-58 at high temperatures in the present study indicated that some heat stable metabolite was present in the CFS of BS-58. Heat stability of antimicrobial protein AsR416 produced by *B. subtilis* was reported at different temperatures (30, 50, 70, and 100°C) by Kong et al. (2018). The heat stable nature of antifungal metabolite might be helpful, when processed industrially as antimicrobial formulation. High temperature will not cause any side effect in the quality of the metabolite. Considerable reductions in antifungal activity of CFS of BS-58 against both the fungal pathogens indicate towards the proteinacious nature of the active metabolite present therein. Tang et al. (2015) also found a reduced activity of AMP after protease action. They suggested that a variety of proteases could hydrolyze the carboxyl-terminal peptide bond of some proteins and destroy the spatial structure of the protein resulting in loss of antifungal activity under certain temperature conditions. However, the specific mechanisms need further verification.

Thin layer chromatography (TLC) performed with different solvent systems suggested the combination of ethyl acetate and methanol (60EA:50 M) as the best solvent system for the separation of antifungal metabolite of *B. subtilis* BS-58 in this study. The rate of migration of a particular compound depends on the absorbent and the solvent system used, therefore, the selection of the suitable solvent system is crucial (Ranjan and Jadeja, 2017). Selection of the solvent system by TLC can reduce the solvent load and thereby provide an accurate solvent system for column chromatography for a better separation and isolation of an antimicrobial metabolite.

Column chromatography is a well-established and widely used technique for the separation and purification of secondary metabolites. In this study, column chromatography revealed F7 (55EA:45 M) as the most active elute responsible for the inhibition of *F. oxysporum* and *R. solani*. Our results get support from the study of Wang et al. (2012), who isolated antifungal metabolite from *B. coagulans* by using TLC guided column chromatography and reported three fractions as active elutes for the suppression of *Phytophthora drechsleri*. Recently, Salazar et al. (2020) extracted antimicrobial metabolite (Macrolactin) from *B. amyloliquefaciens* ELI149 by silica gel column chromatography. The antifungal activity of the active elutes of *Bacillus subtilis* BS-58 in the present study might be attributed to the secretion of secondary metabolite(s).

The mass to charge ratio is measured by LC–MS through the ionization of chemical compounds to generate charged molecules or molecule fragments. In the present study, molecular weight of the antifungal metabolite was determined as 402 Da by its m/z value in LC–MS that corresponds to macrolactin A (402.5 Da). Our results are endorsed by the study of Yuan et al. (2012), who identified antifungal metabolite as macrolactin A produced by *Bacillus amyloliquifaciens* NJN-6 by LC–MS with a molecular weight of 402 Da.

The FT-IR technique is a rapid, time saving method and has been used to identify the compound present in the pure form or in the mixture of various compositions (Kowalczuk and Pitucha, 2019). In our study, different functional groups including alcohol, alkane, carbonyl were detected by the FT-IR analysis of the antifungal metabolite, which were found similar to the functional groups of macrolactin A. Likewise, Devi et al. (2010) identified an antimicrobial compound from *Bacillus licheniformis* SAB1 as 3-phenylpropionic acid by FT-IR analysis.

This study also evidenced the presence of the *mln* gene in *B. subtilis* BS-58 that is responsible for the synthesis of the polyketide of macrolactin group. Macrolactin is a polyketide that is known to inhibit bacterial as well as fungal growth (Kim et al., 2011; Chakraborty et al., 2014; Yuan et al., 2016; Salazar et al., 2020). Earlier, macrolactin type antibiotic was isolated by Yuan et al. (2012) from *B. amyloliquifaciens* NJN-6 and reported significant inhibition of *F. oxysporum* and *Ralastonia solanacearum*. The presence of the *mln* gene in *B. subtilis* BS-58 in the present study, advocates that the metabolite responsible for the effective inhibition of both the fungal pathogens belong to the macrolactin group of antibiotics.

The pot assay revealed the potential of *B. subtilis* BS-58 to suppress the diseases caused by *F. oxysporum* and *R. solani*. This was evident by decreased mortality of *amaranth* seedlings when grown in the soil infested with *F. oxysporum* and *R. solani* and treated with *B. subtilis* BS-58 in comparison to positive control. Data analysis revealed a promising performance of BS-58 for plant growth and disease suppression activities under challenged conditions (T-3), when compared with negative control (T-4; Figure 4C). Seed treatment by BS-58 could increase different growth parameters in *F. oxysporum* amended soil by 27.78 to 117.46 per cent over negative control. However, mortality in seedlings in this treatment was almost 54 per cent less than negative control-1 (T-5). This was encouraging to note that BS-58 showed a comparative performance to affect different growth parameters and disease suppression (except germination) in *F. oxysporum* amended soil (T-3), when compared with positive control (T-4). Seed germination was 23 per cent higher in positive control in comparison to T-3 (Figure 4E).

Similarly, under *R. solani* infested condition (T-6), BS-58 again presented it to be a potential candidate to enhance plant growth by 10.00 to 100 per cent and could reduce the seedling mortality by 43.76 per cent in comparison to negative control-2 (T-8). Again, BS-58 exhibited good performance to affect different growth parameters and disease suppression in *F. oxysporum* amended soil (T-3), when compared with positive control (T-7). However, seed germination was 20 per cent higher and mortality was almost 12 per cent less in positive control in comparison to T-6 (Figure 4F). This inhibition of pathogenic fungi might be due to the production of macrolactin A. Being secretary in nature, this would have spread in rhizosphere and created a non-conducive environment for these pathogenic fungi to grow. Chauhan et al. (2016) reported inhibition of *F. solani* with the reduced percentage incidence of rhizome rot in turmeric (*Curcuma longa* L.) by the treatments of *Bacillus endophyticus* TSH42 and *B. cereus* TSH77. They suggested that the antifungal activity was due to the production of certain antibiotics such as iturin, fungycin and surfactin by this *Bacillus* sp. The above findings endorse that the strong antifungal activity of *B. subtilis* BS-58 in this study was due to the production of macrolactin A. The antifungal activity of macrolactins has previously been reported against several other plant pathogens such as *F. proliferatum*, *Moniliophthora roreri*, *Fusarium* sp., *Aspergillus niger*, *Rhizoctonia* sp. and *A. alternata* (Salazar et al., 2020). Interestingly, mycelial lysis and deformities induced by *B. subtilis* BS-58 in both the pathogens (as observed in SEM photomicrographs) further confirms the promising role of BS-58 in the suppression of fungal diseases. Thus, BS-58 promises to be a potential biocontrol agent for the management of plant diseases.

## 5. Conclusion

The findings of the study reveal that the antifungal activity of *B. subtilis* BS-58 against two destructive phytopathogens is due to the production of macrolactin A. Being extracellular in nature, this would have diffused in rhizosphere and effectively suppressed the fungal infection in *amaranth* seedlings grown in pathogen infested soil. Being native and target specific, such strains under suitable conditions, may result in ample production of antibiotic and greater suppression of the disease.

## Data availability statement

The datasets presented in this study can be found in online repositories. The names of the repository/repositories and accession number(s) can be found in the article/Supplementary material.

## Author contributions

CP performed the experiments and drafted the manuscript. DP conducted *mln* gene amplification and molecular analysis. SD and MG performed the analysis of the experimental data and edited the manuscript. YN and DM conceptualized and supervised the study, and reviewed the manuscript. All authors contributed to the article and approved the submitted version.

## Conflict of interest

The authors declare that the research was conducted in the absence of any commercial or financial relationships that could be construed as a potential conflict of interest.

## Publisher’s note

All claims expressed in this article are solely those of the authors and do not necessarily represent those of their affiliated organizations, or those of the publisher, the editors and the reviewers. Any product that may be evaluated in this article, or claim that may be made by its manufacturer, is not guaranteed or endorsed by the publisher.

## References

[ref1] Ausubel F. M. Brent R. Kingston R. E. Moore D. D. Seidman J. G. Smith J. A.. (1999). Short Protocols in Molecular Biology. John Wiley & Sons, New York, NY.

[ref2] Caulier S. Nannan C. Gillis A. Licciardi F. Bragard C. Mahillon J. (2019). Overview of the antimicrobial compounds produced by members of the *Bacillus subtilis* group. Front. Microbiol. 10:302. doi: 10.3389/fmicb.2019.00302, PMID: 30873135 PMC6401651

[ref3] Chakraborty M. Mahmud N. U. Gupta D. R. Tareq F. S. Shin H. J. Islam T. (2020). Inhibitory effects of linear lipopeptides from a marine *Bacillus subtilis* on the wheat blast fungus *Magnaportheoryzae triticum*. Front. Microbiol. 11:665. doi: 10.3389/fmicb.2020.00665, PMID: 32425899 PMC7203576

[ref4] Chakraborty K. Thilakan B. Raola V. K. (2014). Polyketide family of novel antibacterial 7-O-methyl-5′-hydroxy-3′-heptenoate–macrolactin from seaweed-associated *Bacillus subtilis* MTCC 10403. J. Agric. Food Chem. 62, 12194–12208. doi: 10.1021/jf504845m 25420039

[ref5] Chauhan A. K. Maheshwari D. K. Kim K. Bajpai V. K. (2016). Termitarium-inhabiting *bacillus endophyticus* TSH42 and *Bacillus cereus* TSH77 colonizing *Curcuma longa* L.: isolation, characterization, and evaluation of their biocontrol and plant-growth-promoting activities. Can. J. Microbiol. 62, 880–892. doi: 10.1139/cjm-2016-0249, PMID: 27604298

[ref600] Davis R. Mauer L. J. (2010). “Fourier transform infrared (FT-IR) spectroscopy: a rapid tool for detection and analysis of foodborne pathogenic bacteria,” in Technology and Education Topics in Applied Microbiology and Microbial Biotechnology. Curr. Res. 2, 1582–1594.

[ref6] De la Lastra E. Camacho M. Capote N. (2021). Soil bacteria as potential biological control agents of *fusarium* species associated with asparagus decline syndrome. Appl. Sci. 11:8356. doi: 10.3390/app11188356

[ref7] Deraz S. F. Karlsson E. N. Hedström M. Andersson M. M. Mattiasson B. (2005). Purification and characterisation of acidocin D20079, a bacteriocin produced by *Lactobacillus acidophilus* DSM 20079. J. Biotechnol. 117, 343–354. doi: 10.1016/j.jbiotec.2005.02.005, PMID: 15925717

[ref8] Devi P. Wahidullah S. Rodrigues C. Souza L. D. (2010). The sponge-associated bacterium *Bacillus licheniformis* SAB1: a source of antimicrobial compounds. Mar. Drugs 8, 1203–1212. doi: 10.3390/md8041203, PMID: 20479975 PMC2866483

[ref9] Fortunati E. Mazzaglia A. Balestra G. M. (2019). Sustainable control strategies for plant protection and food packaging sectors by natural substances and novel nanotechnological approaches. J. Sci. Food Agric. 99, 986–1000. doi: 10.1002/jsfa.9341, PMID: 30191564

[ref10] Gomaa E. Z. (2012). Chitinase production by *Bacillus thuringiensis* and *Bacillus licheniformis*: their potential in antifungal biocontrol. J. Microbiol. 50, 103–111. doi: 10.1007/s12275-012-1343-y, PMID: 22367944

[ref11] Guo S. Zhang J. W. Dong L. H. Li X. Asif M. Guo Q. G.. (2019). Fengycin produced by *Bacillus subtilis* NCD-2 is involved in suppression of clubroot on Chinese cabbage. Biol. Control 136:104001. doi: 10.1016/j.biocontrol.2019.104001

[ref12] Hashem A. Tabassum B. Abd_Allah E. F. (2019). *Bacillus subtilis*: a plant-growth promoting rhizobacterium that also impacts biotic stress. Saudi J. Biol. Sci. 26, 1291–1297. doi: 10.1016/j.sjbs.2019.05.004, PMID: 31516360 PMC6734152

[ref13] He L. M. Troiano J. Wang A. Goh K. (2008). “Environmental chemistry, ecotoxicity, and fate of lambda-cyhalothrin,” in Reviews of Environmental Contamination and Toxicology. ed. Whitacre D. M. (New York, NY: Springer), 71–91. 10.1007/978-0-387-77030-7_3 18418954

[ref14] Hussain T. Khan A. A. (2020). *Bacillus subtilis* Hussain T-AMU and its antifungal activity against potato black scurf caused by *Rhizoctonia solani* on seed tubers. Biocat. Agric. Biotechnol. 23:101443. doi: 10.1016/j.bcab.2019.101443

[ref15] Jimtha J. C. Jishma P. Arathy G. B. Anisha C. Radhakrishnan E. K. (2016). Identification of plant growth promoting rhizosphere *bacillus* sp. WG4 antagonistic to *Pythiummyriotylum* and its enhanced antifungal effect in association with *Trichoderma*. J. Soil Sci. Plant Nutri. 16, 578–590. doi: 10.4067/S0718-95162016005000026

[ref16] Kaur P. K. Kaur J. Saini H. S. (2015). Antifungal potential of *Bacillus vallismortis* R2 against different phytopathogenic fungi. Span. J. Agric. Res. 13:e1004. doi: 10.5424/sjar/2015132-6620

[ref17] Kim D. H. Kim H. K. Kim K. M. Kim C. K. Jeong M. H. Ko C. Y.. (2011). Antibacterial activities of macrolactin a and 7-O-succinyl macrolactin a from *Bacillus polyfermenticus* KJS-2 against vancomycin-resistant enterococci and methicillin-resistant *Staphylococcus aureus*. Arch. Pharm. Res. 34, 147–152. doi: 10.1007/s12272-011-0117-0, PMID: 21468926

[ref18] King E. J. Brown M. F. (1983). A technique for preserving aerial fungal structures for scanning electron microscopy. Can. J. Microbiol. 29, 653–658. doi: 10.1139/m83-106, PMID: 6349760

[ref19] Kong X. Yang M. Abbas H. M. Wu J. Li M. Dong W. (2018). Antimicrobial genes from *Allium sativum* and *Pinelliaternata* revealed by a *Bacillus subtilis* expression system. Sci. Rep. 8:14514. doi: 10.1038/s41598-018-32852-x 30266995 PMC6162269

[ref20] Kowalczuk D. Pitucha M. (2019). Application of FTIR method for the assessment of immobilization of active substances in the matrix of biomedical materials. Materials 12:2972. doi: 10.3390/ma12182972, PMID: 31540255 PMC6766236

[ref21] Ku Y. Yang N. Pu P. Mei X. Cao L. Yang X.. (2021). Biocontrolmechanism of *Bacillus subtilis* C3 against bulb rot disease in *Fritillariataipaiensis* P.Y.Li. Front. Microbiol. 12:756329. doi: 10.3389/fmicb.2021.756329, PMID: 34659191 PMC8515143

[ref22] Kumar P. Dubey R. C. Maheshwari D. K. (2012). *Bacillus* strains isolated from rhizosphere showed plant growth promoting and antagonistic activity against phytopathogens. Microbiol. Res. 167, 493–499. doi: 10.1016/j.micres.2012.05.002, PMID: 22677517

[ref23] Kumar S. Suyal D. C. Dhauni N. Bhoriyal M. Goel R. (2014). Relative plant growth promoting potential of Himalayan psychrotolerant *Pseudomonas jesenii* strain MP1 against native *Cicer arietinum* (L.)., *Vigna mungo* (L.) Hepper; *Vigna radiata* (L.)Wilczek., *Cajanus cajan* (L.) mill sp. and *Eleusine coracana* (L.) Garten. Afr. J. Microbiol. Res. 8, 3931–3943.

[ref25] Moyne A. L. Shelby R. Cleveland T. E. Tuzun S. (2001). Bacillomycin D: an iturin with antifungal activity against *Aspergillus flav*us. J. Appl. Microbiol. 90, 622–629. doi: 10.1046/j.1365-2672.2001.01290.x, PMID: 11309075

[ref26] Mulk S. Wahab A. Yasmin H. Mumtaz S. El-Serehy H. A. Khan N.. (2022). Prevalence of wheat associated *Bacillus* spp. and their biocontrol efficacy against fusarium root-rot. Front. Microbiol. 12:798619. doi: 10.3389/fmicb.2021.798619, PMID: 35310393 PMC8927631

[ref27] Negi Y. K. Prabha D. Garg S. K. Kumar J. (2011). Genetic diversity among cold-tolerant fluorescent *Pseudomonas* isolates from Indian Himalayas and their characterization for biocontrol and plant growth-promoting activities. J. Plant Growth Regul. 30, 128–143. doi: 10.1007/s00344-010-9175-7

[ref28] Negi Y. K. Prabha D. Garg S. K. Kumar J. (2017). Biological control of ragi blast disease by chitinase producing fluorescent *Pseudomonas* isolates. Org. Agri. 7, 63–71. doi: 10.1007/s13165-015-0142-2

[ref29] Pandey C. (2018a). Potential of Cold Tolerant Isolates of Bacillus Species for Growth Promotion, Disease Suppression and Yield Enhancement in Grain Amaranthus, Ph.D. Thesis submitted to Gurukul Kangri Vishwavidyalaya, Haridwar (Uttarakhand), India.

[ref30] Pandey C. Bajpai V. K. Negi Y. K. Rather I. A. Maheshwari D. K. (2018b). Effect of plant growth promoting *Bacillus* spp. on nutritional properties of *Amaranthus hypochondriacus* grains. Saudi J. Biol. Sci. 25, 1066–1071. doi: 10.1016/j.sjbs.2018.03.003, PMID: 30174503 PMC6117431

[ref31] Pandey C. Negi Y. K. Maheshwari D. K. Rawat D. Prabha D. (2018c). Potential of native cold tolerant plant growth promoting bacilli to enhance nutrient use efficiency and yield of *Amaranthushypochondriacus*. Plant Soil 428, 307–320. doi: 10.1007/s11104-018-3681-y

[ref32] Pandey C. Prabha D. Negi Y. K. (2018d). “Mycoremediation of common agricultural pesticides,” in Mycoremediation and Environmental Sustainability. ed. Prasad R. (Springer Nature, Switzerland: Springer Publications), 155–179.

[ref33] Ranjan R. Jadeja V. (2017). Isolation, characterization and chromatography based purification of antibacterial compound isolated from rare endophytic actinomycetes *Micrococcus yunnanensis*. J. Pharm. Anal. 7, 343–347. doi: 10.1016/j.jpha.2017.05.001 29404059 PMC5790701

[ref34] Salazar F. Ortiz A. Sansinenea E. (2020). A strong antifungal activity of 7-O-succinyl macrolactin a vs Macrolactin a from *Bacillus amyloliquefaciens* ELI149. Curr. Microbiol. 77, 3409–3413. doi: 10.1007/s00284-020-02200-2, PMID: 32944805

[ref35] Savary S. Willocquet L. Pethybridge S. J. Esker P. McRoberts N. Nelson A. (2019). The global burden of pathogens and pests on major food crops. Nat. Ecol. Evol. 3, 430–439. doi: 10.1038/s41559-018-0793-y, PMID: 30718852

[ref36] Schneider K. Chen X. H. Vater J. Franke P. Nicholson G. Borriss R.. (2007). Macrolactin is the polyketide biosynthesis product of the pks 2 cluster of *Bacillus amyloliquefaciens* FZB42. J. Nat. Prod. 70, 1417–1423. doi: 10.1021/np070070k, PMID: 17844999

[ref37] Shirani M. Raeisi R. Heidari-Soureshjani S. Asadi-Samani M. Luther T. (2017). A review for discovering hepatoprotective herbal drugs with least side effects on kidney. J. Nephropharmacol. 6, 38–48. doi: 10.15171/npj.2017.03

[ref38] Singh P. Singh R. K. Zhou Y. Wang J. Jiang Y. Shen N.. (2022). Unlocking the strength of plant growth promoting *Pseudomonas* in improving crop productivity in normal and challenging environments: a review. J. Plant Interac. 17, 220–238. doi: 10.1080/17429145.2022.2029963

[ref39] Skidmore A. M. Dickinson C. H. (1976). Colony interactions and hyphal interference between *Septoria nodorum*and phylloplane fungi. Transac. Brit. Mycol. Soc. 66, 57–64. doi: 10.1016/S0007-1536(76)80092-7

[ref40] Stein T. (2005). *Bacillus subtilis* antibiotics: structures, syntheses and specific functions. Mol. Microbiol. 56, 845–857. doi: 10.1111/j.1365-2958.2005.04587.x, PMID: 15853875

[ref41] Tang W. Yuan H. Zhang H. Wang L. Qian H. Qi X. (2015). An antimicrobial peptide screened from casein hydrolyzate by *Saccharomyces cerevisiae*cell membrane affinity method. Food Cont. 50, 413–422. doi: 10.1016/j.foodcont.2014.09.030

[ref42] Wang H. Yan Y. Wang J. Zhang H. Qi W. (2012). Production and characterization of antifungal compounds produced by *Lactobacillus plantarum* IMAU10014. PLoS One 7:e29452. doi: 10.1371/journal.pone.0029452, PMID: 22276116 PMC3261852

[ref43] Yuan J. Li B. Zhang N. Waseem R. Shen Q. Huang Q. (2012). Production of bacillomycin-and macrolactin-type antibiotics by *Bacillus amyloliquefaciens* NJN-6 for suppressing soilborne plant pathogens. J. Agric. Food Chem. 60, 2976–2981. doi: 10.1021/jf204868z, PMID: 22385216

[ref44] Yuan J. Zhao M. Li R. Huang Q. Rensing C. Raza W.. (2016). Antibacterial compounds-macrolactin alters the soil bacterial community and abundance of the gene encoding PKS. Front. Microbiol. 7:1904. doi: 10.3389/fmicb.2016.01904, PMID: 27965639 PMC5126139

[ref45] Zhang T. Shi Z. Q. Hu L. B. Cheng L. G. Wang F. (2008). Antifungal compounds from *Bacillus subtilis* B-FS06 inhibiting the growth of *Aspergillus flavus*. World J. Microbiol. Biotechnol. 24, 783–788. doi: 10.1007/s11274-007-9533-1

[ref46] Zhu J. Tan T. Shen A. Yang X. Yu Y. Gao C.. (2020). Biocontrol potential of *Bacillus subtilis* IBFCBF-4 against fusarium wilt of watermelon. J. Plant Pathol. 102, 433–441. doi: 10.1007/s42161-019-00457-6

[ref47] Zhu F. Wang J. Jia Y. Tian C. Zhao D. Wu X.. (2021). *Bacillus subtilis* GB519 promotes rice growth and reduces the damagescaused by rice blast fungus *Magnaportheoryzae*. PhytoFront. 1, 330–338. doi: 10.1094/PHYTOFR-12-20-0041-R

