# Supplementary material for: Macrolactin A mediated biocontrol of Fusarium oxysporum and Rhizoctonia solani infestation on Amaranthus hypochondriacus by Bacillus subtilis BS-58
Source: Front Microbiol. 2023 Feb 23;14:1105849. doi: 10.3389/fmicb.2023.1105849 (PMC10032343; doi:10.3389/fmicb.2023.1105849)
Supplement: Supplementary file 1 [file Table_1.DOCX]

**Appendix A**

**Supplementary Table S1:** Solvent system for thin layer chromatography

| **Solvent system** | **Ratio** |
| --- | --- |
| Chloroform : methanol : acetic acid | 6:3:1 |
| Chloroform : methanol : acetic acid | 65:34:1 |
| Chloroform : methanol : acetic acid | 85:15+4 drops |
| Ethyl acetate : chloroform | 5:5 |
| Ethyl acetate : chloroform | 6:4 |
| Ethyl acetate : chloroform | 7:3 |
| Ethyl acetate : chloroform | 8:2 |
| Ethyl acetate : chloroform | 9:1 |
| Ethyl acetate : chloroform : acetic acid few drops | 7:3 |
| Chloroform : methanol : acetic acid | 90:8:2 |
| Chloroform : methanol : water | 64:24:4 |
| Ethyl acetate : chloroform : acetic acid few drops | 8:2 |
| Ethyl acetate : chloroform : methanol | 5:3:2 |
| Ethyl acetate : methanol | 60:40 |
| Ethyl acetate : methanol | 70:30 |
| Ethyl acetate : methanol | 80:20 |
| Ethyl acetate : methanol : water | 5:4:1 |

**Supplementary Table S2:** Gene Sequence *Bacillus subtilis* BS-58

| **Primers used** | **MLN-C1:** ATGCTGTTGCAGGACATAGTC and  **MLN-C2:** TAGTCAGAATGTTTCCAGGACC (Schneider et al. 2007) |
| --- | --- |
| **Sequence retrieved** | CATAGTCTGGGTGAATATACTGCGCTTTTTGCGGCCGGCGTTTTTAATTTTGAAACAGGTCTCCGGCTGGTTGCTAAAAGAGGCGAGTTAATGAGTGAGGCTTCCGAGGGCGGAATGGCTGCCGTGATCGGACTGGATGAGCATCACATAAAAAAAATACTTCAGAAGTATGAATTCGGCCAAATAGATATCGCCAATTACAATACATCTTCACAAATTGTGATAGCAGGGGCTGCAGATGAGATAAAGCGGGCAGCCTCTTTTTTTGAAAAAGAAGGGGCGAAAGCTTATATCGTATTGCAAGTCGGCGGAGCTTTTCATTCTCGTTTCATGGAAAGTGCACAGCGTGAATTTGCGGAATTTATCGAGGAGTTTCATTTTTCTGAATTGAATTTCCCGGTCATATCTAACTATACGGCAAGACCTTATAAACAAGAAGATATCAAACGGAATTTAATTGAACAAATAACAAACTCGGTGAAATGGACTGAAAGCATCAGGTATCTAATGGGACAAGGCGTAACTGTGTTTGAAGAGATCGGTCCTGGAACTTT |
| **Accession number** | **MT726941** |
